# Later achievement of infant motor milestones is related to lower levels of physical activity during childhood: the GECKO Drenthe cohort

**DOI:** 10.1186/s12887-019-1784-0

**Published:** 2019-10-28

**Authors:** Silvia I. Brouwer, Ronald P. Stolk, Eva Corpeleijn

**Affiliations:** 1 Hanze University of Applied Sciences, Institute of Sportstudies, Zernikeplein 17, 9747 AS Groningen, The Netherlands; 2 Department of Epidemilogy, University Medical Center Groningen, Hanzeplein 1, 9713 GZ Groningen, The Netherlands

**Keywords:** Infant, Motor development, Obesity, Physical activity

## Abstract

**Background:**

The aim of this study is to investigate whether age of infant motor milestone achievement is related to levels of physical activity (PA), weight status and blood pressure at age 4–7 years of age.

**Methods:**

In the Dutch GECKO (Groningen Expert Center of Kids with Obesity) Drenthe cohort, the age of achieving the motor milestone ‘walking without support’ was reported by parents. Weight status and blood pressure were assessed by trained health nurses and PA was measured using the Actigraph GT3X between age 4 and 7 years.

**Results:**

Adjusted for children’s age, sex and the mother’s education level, infants who achieved walking without support at a later age, spent more time in sedentary behaviour during childhood and less time in moderate-to-vigorous PA. Later motor milestones achievement was not related to higher BMI Z-score, waist circumference Z-score, diastolic or systolic blood pressure.

**Conclusion:**

The results of this study indicate that a later age of achieving motor milestone within the normal range have a weak relation to lower PA levels at later age. It is not likely that this will have consequences for weight status or blood pressure at 4–7 years of age.

## Background

The importance of physical activity (PA) in the early years of life has been documented for a broad spectrum of health benefits, for example improved fitness, motor skill competence, cognitive development, psychosocial health, and cardiometabolic health [1, 2]. PA tracks from early childhood into middle childhood [3] and into adulthood [4]. Identifying early life factors that influence childhood PA may help to increase PA in later life. In the long run, this might be important for public health promotion strategies.

One early life factor associated with childhood PA is motor skill competence [5–7]. In children motor skill competence is often assessed by measuring fundamental movement skills like jumping, hopping, running, and throwing. Several cross-sectional studies have shown that children aged 6 to 12 years who have lower levels of motor skill competence tests have lower levels of objectively measured PA and higher levels of sedentary behavior (SB) [6–8] compared to children who have higher levels of motor skill competence. However, the direction of the association is not clear and the relationship may in fact be reciprocal [9] and dependent on age. On the one hand, as children develop, adequate motor skill competence is of importance for participation in PA [10]. On the other hand, engagement in PA at young age may be important for the development of motor skill competence [11]. Prospective studies are needed but evidence for the direction of this association is scarce, especially in young populations [12]. The association between infant motor skill competence and objectively measured PA later in childhood has only been studied in a population of 2 year-old children [13] and in a 11–12 year-old population [14]. Since only one of the two studies showed a significant association, more clarity is needed whether infants who *develop their motor skills later*, but within the normal range, are less physically active during childhood.

Since PA levels have dropped during the last decennia [15] a focus on infants motor skill competence might help to target inactivity during childhood. In addition to PA, low levels of motor skill competence has also been related to the development of overweight, obesity and blood pressure in children [16–18]. Since low levels of motor skill competence may be related to the development of obesity, the question rises whether this is mediated by lower levels of PA.

To gain more insight in the relation between motor skill competence and PA, we will investigate whether later achievement of the motor milestone “walking without support” is related to lower levels of PA, and more time spent in SB at later age (4–7 years). Second, we will investigate whether later achievement of the motor milestones “walking without support” is related to higher weight status and blood pressure, and if so, whether this is mediated by PA.

## Methods

### Participants

The GECKO (Groningen Expert Center of Kids with Obesity) Drenthe birth cohort is a population-based birth cohort that has been designed to study the determinants and development of childhood weight status. All parents of children born between April 2006 and April 2007 in the province of Drenthe in the Netherlands were invited to participate in the study. Further details regarding the study design, recruitment and study procedures have been published elsewhere [19].

### Child characteristics

Gestational age (GA) and educational level of the mother (low/middle education or higher vocational education) was self-reported. Educational level was reported since it is part of socioeconomic status which is associated with motor skill competence [16]. Anthropometry of children was measured by trained nurses from Youth Health Care according to a standardized protocol when children were 4–7 years old. Weight was measured in light clothing using an electronic scale with digital reading, and recorded to the nearest 0.1 kg. Height was assessed using a stadiometer and recorded to the nearest 0.1 cm. Waist circumference (WC) was measured twice using a standard tape midway between the lowest rib and the top of the iliac crest at gentle expiration in standing position to the nearest 0.1 cm. When the two measurements differed more than 1 cm a third measurement was done. BMI was calculated as weight (kg) divided by height squared (m). Gender and age-specific BMI Z-scores and WC Z-scores were calculated using the Dutch growth analyser software, version 3.5 based on 1997 reference data (https://growthanalyser.org/software/growth-analyser-rct/). Systolic (SBP) and diastolic blood pressure (DBP) (mmHg) were measured using a digital automatic blood pressure monitor (M3 intellisense™, OMRON healthcare Co. Japan) with the smallest cuff. The cuff was placed on the left arm of the relaxed and seated child and the measurements were repeated up to 3 times at one-minute intervals. SBP and DPB Z-scores were calculated considering the child’s exact age, height and gender using the fourth report on the diagnosis, evaluation and treatment of high blood pressure in children and adolescents as a reference [20].

### Motor skill competence and physical activity

Motor skill competence in infants is often assessed by the age of achievement of motor milestones (like sitting, crawling, standing and walking with or without support). We used the motor milestone ‘walking without support’ since the achievement of different motor milestones follows a fixed sequence for sitting without support, standing with assistance, walking with assistance, standing alone and walking alone) and therefore suggest a correlation [21, 22]. Walking without support is considered to be universal, fundamental to the acquisition of self-sufficient erect locomotion, and simple to test and evaluate [23, 24]. The question ‘at how many months did your child walk without support for the first time’ was assessed after the child Youth Health Care visit at 18 months via parents who filled in the surveys. Infants who had not achieved the milestone of walking without support by age 18 months were not included in the analyses. Previous research has shown that retrospective surveys completed by the mother on the infant’s gross motor milestones are a reliable source of data [25] although a bias towards earlier dates of achievement are likely [WHO; reliability). Maternal recall and report of infant’s milestone achievement has been used in several other studies [26, 27]. PA was assessed between 2009 and 2013 when the children were between 4 and 7 years old using the ActiGraph GT3X accelerometer (ActiGraph, Pensacola, FL). The ActiGraph has been shown to be a reliable and valid device for measuring PA volume and intensity in young children [28]. Parents were instructed to have their child wear the ActiGraph on the iliac crest on the right hip with an elastic belt for four consecutive days, including at least one weekend day, during all waking hours except while bathing or swimming. Data were collected at a frequency of 30 Hz. All children who recorded a wearing time ≥ 840 min/day (14 h/day) were checked manually for sleeping time and data were corrected if necessary. ActiGraph non-wearing time was classified as a period of a minimum of 90 min without any observed counts [29]. The cut-off points recommended by Butte et al. [30] were used to calculate time spent in SB (< 240 counts per minute (cpm), light PA (LPA) (241–2120 cpm), moderate PA (MPA) (2121–4450 cpm), and vigorous PA (VPA) (> 4450 cpm). The data were analysed in 15-s epochs [31]. Mean SB, LPA, MPA, and VPA were calculated per child using all days with wear time ≥ 600 min/day. Moderate to vigorous PA (MVPA) was calculated by summing up the time spent in MPA and VPA. Adherence to the Dutch healthy exercise norm was defined as ≥60 min of MVPA per day. To be included in the analysis in this study, the accelerometer had to be worn for at least 600 min/day for at least 3 days.

### Statistics

Data were analysed using SPSS 23.0. BMI, MPA, VPA, MVPA and total PA were Ln transformed because of skewedness. A two-tailed Student’s t-test was used to test for gender differences. Means ± standard deviations or the median (25th, 75th percentile) are presented. To test whether later achievement of the motor milestones “walking without support” is related to lower levels of PA, and more time spent in SB at later age (4–7 years) we used separate multiple linear regression models to examine associations of age of achievement with each of the PA outcomes (SB, LPA, MVPA and total PA), as continuous variables. We first ran model 1 for unadjusted analyses examining the relationship between individual motor milestones and each PA outcome. In model 2 we included exact age of assessment of PA, sex and maternal educational level as covariates. When testing whether later achievement of the motor milestones “walking without support” is related to higher weight status and blood pressure we used the same model with BMI Z-scores, WC Z-score, DBP Z-score and SBP Z-score as the outcome. Analysis on DBP Z-score and SBP Z-score were additionally adjusted for height. To test whether a possible relation between later achievement of the motor milestones “walking without support” and higher weight status or blood pressure is mediated by PA, we added each of the PA outcomes to the model. Because data of MVPA and total PA were Ln transformed the β’s do not reflect actual minutes/day MPVA or total PA but reflect Ln transformed results. By filling in the regression analyses we calculated the Ln MVPA for different ages of motor milestones achievement (in months). The amount of minutes/day MVPA corresponding to the outcome of the regression were looked up in the original file to translate it into meaningful data.

## Results

**Fig. 1:**
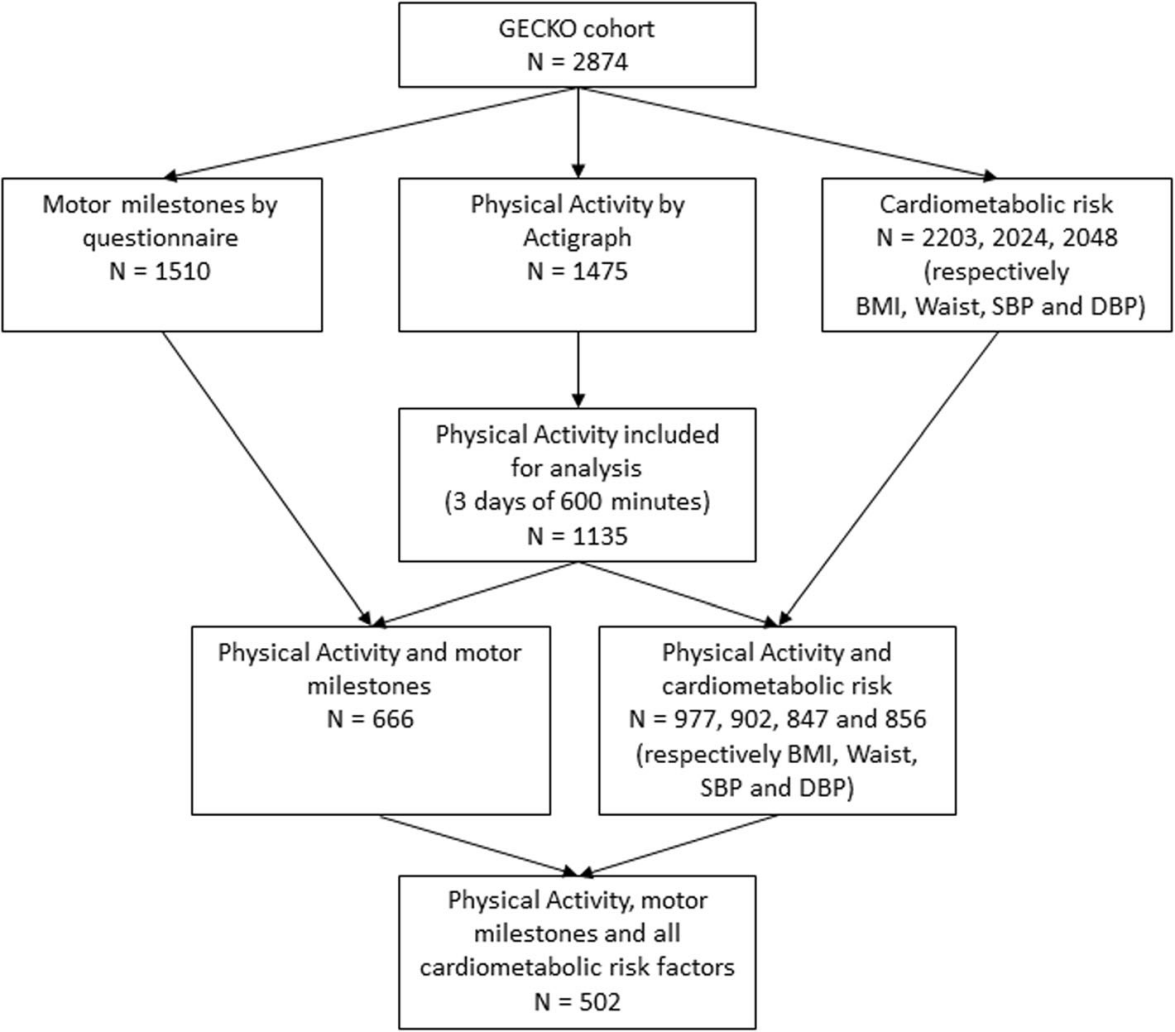
Flowchart of the participants. All participants were recruited from the GECKO Drenthe birth cohort (babies born between April 1st 2006 and April 1st 2007 in Drenthe, Netherlands) and measured for PA between 2009 and 2012 when aged 4–7 years

In total, parents of 2997 children expressed the intention to participate in the study, 2874 of whom actively participated. The flowchart (Fig. 1) shows the GECKO cohort with available data for motor milestones achievement, PA and cardiometabolic risk. The questionnaire for motor milestones achievement was handed out to parents who visited the Well Baby Clinic and for logistic reasons not all parents who actively participated in the study received a questionnaire. The questionnaire was filled in by 1672 parents. From 1672 children 7% (*n* = 117) were not able to walk without support at 18 months, 2% (*n* = 39) of the parents filled in the questionnaire but didn’t fill in the question with how many months their child was able to walk without support, and < 1% (*n* = 5) filled in implausible data (walking before age 5 months) and were therefore excluded from analyses. The parents of 2276 children were contacted for PA measurements and 1475 of these children were measured for PA with an ActiGraph GT3X accelerometer (ActiGraph, Pensacola, FL) between the age of 4 and 7 years. Of those, 1135 children had valid Actigraph data. There were 666 children with data for both motor milestones and PA and there were in total 502 children who had complete data for motor milestones, PA and cardiometabolic risk.

To check for bias in the study population for parents who did or did not report the age of achievement of the motor milestone “walking without support”, children with data for motor milestones achievement were compared to children without data for motor milestone achievement. Children without data for motor milestone achievement spent 6 min per day more in SB compared to children with data on motor milestone achievement (*p* = 0.04). No differences were found in MPA, VPA, MVPA and total PA between these groups. Furthermore, no differences were found between SB and PA levels of children with or without data for BMI, waist, DBP and SBP. To check for a bias the other way around, we tested whether the age of motor milestone achievement differed between children with data Actigraph data and without Actigraph data. There were no differences between those groups (*p* = 0.96).

Table 1 presents the baseline characteristics of the population. Children were able to walk without support at the age of 14.1 ± 1.9 months. This age was comparable to the normative sample of the World Health Organization’s Multicentre Growth Reference Study [17]. Children born with a shorter GA achieved their moment of walking later compared to children with longer GA (r^2^–0.15; *P* < 0.001). Children’s PA was assessed on average at 5.8 years. About 50% of children adhere to the Dutch guidelines for PA.

**Table 1:** Characteristics of the GECKO Drenthe cohort

	Total	Boys	Girls
Child characteristics			
Gestational age (weeks)(1089)	39.9 ± 1.6	39.8 ± 1.6	39.9 ± 1.5
Age of assessment (years)(1135)	5.8 ± 0.3	5.9 ± 0.3	5.8 ± 0.3
Height (cm) (977)	118.5 ± 5.1	118.8 ± 5.1	118.2 ± 5.0
Weight (kg) (977)	22.5 ± 3.0	22.5 ± 2.8	22.4 ± 3.2
BMI (kg/m^2^) (977)	15.8 (15.1, 16.7)	15.8 (15.1, 16.6)	15.7 (15.0, 16.7)
BMI Z-score (SD) (977)	0.2 ± 0.8	0.2 ± 0.7	0.2 ± 0.8
Waist (cm) (902)	54.6 ± 4.3	54.7 ± 4.4	54.5 ± 4.2
Waist Z-score (SD) (902)	0.4 ± 1.0	0.3 ± 1.0	0.4 ± 0.9*
DBP (mmHg) (856)	62.0 ± 8.3	60.7 ± 7.9	62.4 ± 7.5*
DBP Z-score (SD) (856)	0.3 ± 0.7	0.1 ± 0.7	0.5 ± 0.7**
SBP (mmHg) (847)	103.3 ± 9.6	103.8 ± 8.5	103.3 ± 8.5
SBP Z-score (SD) (847)	0.6 ± 0.8	0.5 ± 0.8	0.7 ± 0.8**
Motor milestones and Physical activity			
Age ‘walking without support’ (months)(666)	14.1 ± 1.9 (7.0–19.0) 14.0 (13.0–15.0)	14.1 ± 1.9 (9.0–19.0) 14.0 (13.0–15.0)	14.0 ± 1.9 (7.0–19.0) 14.0 (13.0–15.0)
Age of assessment (years)(1135)	5.6 ± 0.8	5.7 ± 0.8	5.6 ± 0.8
SB (min/day) (1135)	373.0 ± 55.3	367.5 ± 54.4	379.0 ± 55.6**
LPA (min/day) (1135)	264.9 ± 38.1	264.9 ± 36.5	264.8 ± 39.7
MPA (min/day) (1135)	43.8 (34.7, 54.9)	47.2 (39.3, 59.8)	40.0 (30.9, 48.8)**
VPA (min/day) (1135)	16.7 (11.3, 24.3)	18.6 (12.8, 26.4)	14.7 (10.2, 22.0)**
MVPA (min/day) (1135)	61.3 (47.8, 80.0)	68.1 (53.2, 85.6)	54.5 (42.0, 71.2)**
Total PA (cpm) (1135)	1319.5 (1140.9, 1522.1)	1362.6 (1202.9, 1585.1)	1250.1 (1079.0, 1457.3)**

Data are presented as means ± sd (with minimum and maximum for age ‘walking without support’) or median (25th, 75th percentile) and number of participants (n). *GA* gestational age, *DBP* diastolic blood pressure, *SBP* systolic blood pressure, *SB* sedentary behaviour, *LPA* light physical activity, *MPA* moderate physical activity, *VPA* vigorous physical activity, *MVPA* moderate to vigorous physical activity, *Total PA* total physical activity; *significant gender differences *p* < 0.05; **p < 0.01

The associations between motor milestone achievement and PA are presented in Table 2. Model 1 shows that later age of achieving moment of walking was associated with higher SB (β = 3.59 [95%CI: 1.37; 5.82]), lower LPA (− 2.11 [− 3.65; − 0.57]), lower Ln MVPA (− 0.03 [− 0.05; − 0.02]) and lower Ln total PA (− 0.02 [− 0.03; − 0.01]). When adjusting for sex, actual age of the child and mother’s education level, the associations between motor milestone achievement and PA remain significant for all PA levels: SB (2.73 [0.60; 4.86]), Ln MVPA (− 0.03 [− 0.05; − 0.02]) and Ln total PA (− 0.02 [− 0.02; − 0.01]) except for LPA (− 1.40 [− 2.85; 0.06]). This means that infants who achieve their motor milestone at a later age spend more time in SB and less time in MVPA, and have lower levels of total PA during childhood. For example, an infant who walks with the age of 12 months spends on average 64.7 min per day in MVPA while an infant who walks with 16 months spends on average 56.7 min per day in MVPA.

**Table 2:** Later achievement of motor milestone ‘walking without support’ was related to lower levels of childhood physical activity in the GECKO Drenthe cohort

	SB (min/day)			LPA (min/day)			Ln MVPA (min/day)			Ln Total PA (min/day)		
	β	B	95% CI	β	B	95% CI	β	B	95% CI	β	B	95% CI
Model 1												
Motor milestone achievement (months)	0.124	3.594	1.369; 5.819	−0.105	−2.109	−3.647; − 0.571	−0.156	− 0.032	− 0.047; − 0.016	−0.140	− 0.016	− 0.025; − 0.007
Model 2												
Motor milestone achievement (months)	0.094	2.726	0.595; 4.856	−0.070	−1.394	− 2.846; 0.058	−0.160	− 0.033	− 0.048; − 0.018	−0.131	− 0.015	− 0.024; − 0.007

Data is presented as standardized beta coefficients (β), unstandardized B and 95% confidence interval; *SB* sedentary behaviour (min/day), *LPA* light physical activity (min/day), *MVPA* moderate-to-vigorous physical activity (min/day), *Total PA* Total physical activity. MVPA and Total PA were Ln transformed because of skewedness. Model 1 shows the unadjusted association between motor milestone achievement and different levels of PA. Model 2 shows the association between motor milestone achievement and different levels of PA adjusting for sex, actual age of the child and maternal education level

Second, the age of achieving ‘walking without support’ was not related to relevant health outcomes. Motor milestone achievement was not associated to BMI Z-score (− 0.01 [− 0.05, 0.02]) or WC Z-score (− 0.01 [− 0.06, 0.03]), nor to DBP Z-score (− 0.02 [− 0.06, 0.01]) or SBP Z-score (− 0.02 [− 0.06, 0.01]). Since motor milestone achievement was not related to most health outcomes, we did not further investigate whether this association was mediated by the level of PA.

## Discussion

In this study, we show that later achievement of the motor milestone ‘walking without support’ is related to lower PA later in childhood. We also show that later achievement of the motor milestone ‘walking without support’ does not seem to have consequences for health outcomes like BMI, WC of blood pressure at the age of 4–7 years.

This study showed that children who achieve their motor milestone later are less physically active during childhood. To our knowledge, and as reviewed by Oglund et al. [12], the associations between infant motor skill competence and objectively measured PA later in childhood have only been studied in a population of 2 year-old children [10] and in a 11–12 year-old population [14]. These studies are however well in line with the present findings. The Avon Longitudinal Study of Parents And Children (ALSPAC) [14] showed that infants with lower maternally reported motor skill competence at 6 months had lower levels of objectively measured PA in children aged 11–12 years. A trend towards significance (*p* < 0.1) was visible for achieving motor milestones at age 1 and lower levels of objectively measured PA at age 2 [13]. Also studies using questionnaire based estimates of PA in children point into the same direction since older age at walking was associated with lower self-reported weekly sport participation in youth aged 14 years [27].

The question rises whether differences in motor skill competence are relevant to differences in PA in children. As explained in the results, an infant who walks without support at the age of 14 months spends on average 4 min less in MVPA and 7 more minutes in SB per day compared to an infant who walks without support at 12 months. This means that a child is 28 min (7 day/week 4 min) per week less active in MVPA when motor milestones are achieved 2 months later. These 28 min MVPA per week seem relevant since it has been demonstrated in observational studies that there is a dose-response relationship between PA and health [32]. Participating in as little as 2 or 3 h of MVPA per week is already associated with health benefits. Therefore, identifying early life determinants of young people’s PA generates meaningful knowledge for future public health interventions, since PA tracks from childhood to adolescence, and then on to adulthood [4, 33]. Stimulating motor skill competence in early life may add to the potential strategies available to enhance MVPA. However, enhancing MVPA is not particularly easy, taking into account that the outcomes of most multi-level interventions show minimal to no increases in PA. The review by Ling et al. [34] shows that most multi-level interventions with objectively measured PA in young children do not find an effect on PA. From the 20 studies included in the review, just 3 studies found an increase in MVPA, 3 studies found an increase in total PA and 2 studies found a decrease in SB.

Since we found that infants who achieve their motor milestone “walking without support” at a later age, although within the normal range, had lower PA levels during childhood, we were asking ourselves if variations in achieving motor milestones could be related to variations in health outcomes. We would expect that children with somewhat later motor milestone achievement and lower PA could have a higher BMI and WC, based on evidence that higher levels of PA may lead to healthier outcomes in cross sectional as well as longitudinal studies [35, 36]. However, in our study infants who achieve their moment of walking without support later did not have higher BMI or WC during childhood. How can we explain the absence of an association between infant motor skill competence and childhood BMI in our study? In contrast to our findings, there is ample evidence for a relation between motor skill competence and overweight, mostly from cross-sectional studies [16, 35]. Most but not all of these cross sectional studies in children and adolescents show that children and adolescents who have lower levels of motor skill competence have a higher BMI. Also in infants, delays in motor skill competence were found more often in overweight compared to normal weight infants [37–39]. These cross-sectional studies cannot account for reverse causation. Motor skill competence may limit PA and thereby increase the risk for overweight, but the other way around, overweight may limit PA and therefore motor skills may be practised less. Prospective studies are necessary to gain more insight. Two prospective studies showed no relationship between delayed motor milestone achievement and BMI at age 3 and 5, but did find an association with higher sum of skinfolds at 3 years of age [26, 40]. Furthermore, a randomized controlled trial testing an early life activity stimulation program delivered to parents in Well Baby Clinics showed benefits on adiposity at the age of 2.5 years [41]. Therefore it is possible that there is an association between delayed motor milestone achievement and childhood body composition when other measures of adiposity are used. In general, the association between motor skill competence and adiposity may be bidirectional, and the effects are likely to be small or absent.

The strength of the study is the objective measure for PA in a relative young population and the large size of the population, although in the combination between motor skill competence and PA many motor skill competence data were missing. Furthermore, our study showed that the associations between motor skill competence and PA were consistently present in all PA behaviours except for LPA. The limitations of the study are the use of only motor milestone “walking without support” as a measure for motor skill competence, since walking is also PA. Therefore it could also be that the association we measure is tracking of PA. We can also not exclude that children who walk earlier may have been stimulated more by parents. We have shown before that activity levels of the parents are related to activity levels of the children [42]. Only infants who reached their motor milestone “walking without support” within the normal range of development, were included in this study. Therefore, it is not possible to make any statements about clinical motor delays.

## Conclusion

The results of this study indicate that a later age of achieving motor milestone within the normal range have a weak relation to lower PA levels at later age. It is not likely that this will have consequences for weight status or blood pressure at 4–7 years of age.

## Data Availability

The GECKO Drenthe cohort is registered at www.birthcohorts.net. Requests for data sharing can be addressed here. The datasets used and/or analysed during the current study are available from the corresponding author on reasonable request.

## Abbreviations

BMI: Body Mass Index
GA: Gestational age
GECKO: Groningen Expert Center of Kids with Obesity
IBM: International Business Machines Corporation
LPA: Light Physical Activity
MVPA: Moderate to Vigorous Physical Activity
PA: Physical Activity
SB: Sedentary behavior
SES: Socioeconomic Status
SPSS: Statistical Packages in Social Science
VPA: Vigorous Physical Activity
